# Editorial: Nystagmus in vestibular and cerebellar disorders

**DOI:** 10.3389/fneur.2023.1289354

**Published:** 2023-09-20

**Authors:** Tzu-Pu Chang, Amir Kheradmand, Ji-Soo Kim, Yoshiko Kojima, Mario U. Manto

**Affiliations:** ^1^Department of Neurology/Neuro-medical Scientific Center, Taichung Tzu Chi Hospital, Buddhist Tzu Chi Medical Foundation, Taichung, Taiwan; ^2^Department of Neurology, School of Medicine, Tzu Chi University, Hualien, Taiwan; ^3^Department of Neurology, Johns Hopkins, Baltimore, MD, United States; ^4^Department of Neurolgy, Seoul National University College of Medicine, Seoul National University Bundang Hospital, Seongnam, South Korea; ^5^Department of Otolaryngology – Head and Neck Surgery, Washington National Primate Research Center, University of Washington, Seattle, WA, United States; ^6^Service des Neurosciences, Université de Mons, Mons, Belgium

**Keywords:** cerebellum, vestibular, head impulse test (HIT), VOR, reflex

The vestibular system and cerebellum are richly interconnected to fine tune eye movements and the vestibulo-ocular reflex (VOR) to maintain images steady on the fovea. Within this network, different aspects of VOR function including nystagmus are valuable to study ocular motor control, examine key structural–functional correlations, and improve clinical diagnosis. The VOR is sensitive to head rotation, translation, or tilt using signals from the receptors in the inner ear. During rotation, the VOR can stabilize the gaze position accurately even at high angular velocities. In clinical practice, the most useful test to evaluate rotational VOR dysfunction is head impulse test (HIT). Since its initial description in 1988 (1), the HIT has entered into clinical routine and the advent of video HIT (vHIT) has increased its interest for research and clinical applications. With recent advances in video-oculography, it is now also possible to measure the otolith-ocular function at the bedside using the video ocular counter-roll (vOCR) test (2).

The vHIT can be performed both in the horizontal and vertical planes of the semicircular canals (3). It is often considered that the accurate application of the vHIT is limited to experts. In a prospective cohort study, Korda et al. assessed the learning curve in novices. An instructional video was used to show optimal head impulses by pointing out fast but small-angle head movements at a correct plane, sufficient distance from the fixation point, and correct positioning of the examiner's hands. This study shows that vHIT was achievable by non-experts after a short learning curve of 180 trials. Novices learned to perform vHIT very fast, provided that they received instruction and feedback from an experienced examiner. It appears that video instructions alone were not sufficient to properly learn the vHIT task. The most common pitfalls in recording vHIT were low head acceleration, large head overshoots impacting on the camera view, and extended excursion angles of head movements.

In a study by Kojima et al., compensatory saccades were examined in vestibular impaired monkeys. The monkeys were required to fixate on a target and the head was rapidly and unexpectedly rotated to stimulate the horizontal semi-circular canals. Similar to human subjects, monkeys made compensatory saccades. These catch-up saccades were compared with the saccades made to follow a moving visual target. The shortest latency of the visual saccades was 250 ms, which indicates that it requires at least 250 ms to induce saccades by a visual signal. The latency of some compensatory saccades during the HIT was shorter than 250 ms, suggesting that they were not induced visually. The peak velocity of the short latency saccades was significantly lower than that of longer latency saccades. The peak velocity of the longer latency saccades was closer to that of visually guided saccades induced by a stepping target. These results are consistent with the studies of overt and covert saccades in human patients.

The early diagnosis of an acute vestibular syndrome is challenging and has major implications. In particular, sorting the differential diagnosis between vestibular neuritis (VN) and strokes in the posterior fossa is a key-step. The reduced VOR gain and the presence of corrective saccades in vHIT are typically classified as an indicator of peripheral vestibulopathy. Regarding central nervous system (CNS) lesions, several patterns have been reported. Lesions involving the vestibular nuclei, nucleus prepositus hypoglossi, or flocculus are associated with reduced gains unilaterally or bilaterally. Cerebellar lesions are associated with hypoactive or hyperactive VOR gains during the vHIT for the vertical canals (4). Among posterior fossa lesions, the infarcts within the posterior inferior cerebellar artery (PICA) territory may show normal or subtle cerebellar signs (5), and thus can be particularly difficult to distinguish from VN. Nam et al. compared vHIT findings between PICA strokes and VN. Patients with PICA infarcts display bilaterally reduced VOR gains for the horizontal and posterior semicircular canals. The receiver operating characteristic curve demonstrates that the asymmetry in the corrective saccades (CSs) amplitudes and asymmetry in VOR gains were excellent parameters to distinguish PICA strokes from VN. PICA strokes could be diagnosed when the CS amplitude of the ipsilesional horizontal semicircular canal (HC) was below 2.55 (sensitivity at 87% and specificity at 88%) or when the VOR gain for the ipsilesional HC was above 0.71 (sensitivity of 88% and specificity of 87%). Overall, it appears that quantification of the corrective saccades provides useful information for the differential diagnosis between peripheral and central vestibulopathies.

Dizziness and unsteadiness are typical complaints in vestibular disorders. Figtree et al. have studied the prevalence of benign paroxysmal positional vertigo (BPPV), and peripheral/central vestibular function in patients (age >50 years) who experienced dizziness within the past year. One hundred and ninety-three patients were tested with a following test battery: clinical HIT (cHIT) and vHIT overshoot to test the high-frequency vestibular organ function, the head thrust dynamic visual acuity test to assess high-frequency visual-stability, the dizziness handicap inventory to quantify the impact of dizziness, and a sinusoidal and unidirectional rotational chair testing to evaluate low- to mid-frequency peripheral and central vestibular function. BPPV, and peripheral or central vestibular hypofunction were found in one third of patients. More than half (57%) of these with a likely vestibular cause had BPPV. This study highlights that one vestibular test alone is unsatisfactory to determine the cause of dizziness.

In a study by Maruta a series of repetitive motions was performed in rabbits that involved a complex combination of roll, pitch, and yaw movements in a head-based reference frame. Eye movements in three dimensions were sampled during the conditioning stimulus as well as during the test stimuli before and up to several days after conditioning. While rabbits were conditioning to combined roll and rotation, the roll component of the VOR was compensatory to the oscillation, but the pitch component was not. After conditioning, simple side-to-side rolling induced an ocular response in the opposite direction during conditioning, indicating a maladaptive change. The impact of conditioning appeared to be partially retained even after 1 week, and could be partially reversed or cumulated depending on the rotation direction with subsequent conditionings. These observations can be valuable to study adaptation within the vestibular cerebellar circuits and potential use of a rabbit model to examine the role of these circuits in disorders such as mal de débarquement syndrome.

In conclusion, the goal of this Research Topic was to gather studies from clinicians and scientists on nystagmus and related mechanisms within the vestibular system and cerebellar regions that control ocular motor functions. These studies included (i) quantitative methods for recording and analysis of nystagmus, (ii) mechanism of gaze stabilization related to vestibular and cerebellar functions and their pathophysiology, and (III) patterns of nystagmus for clinical diagnosis of vestibular and cerebellar disorders. Studies of the VOR are particularly relevant in clinical settings.

## Author contributions

MM: Writing—original draft, Writing—review and editing. T-PC: Writing—review and editing. AK: Writing—review and editing. J-SK: Writing—review and editing. YK: Writing—review and editing.
